# Supplementary figures and images for: Biochemical analysis, photosynthetic gene (psbA) down–regulation, and in silico receptor prediction in weeds in response to exogenous application of phenolic acids and their analogs
Source: PLoS One. 2023 Mar 23;18(3):e0277146. doi: 10.1371/journal.pone.0277146 (PMC10035924; doi:10.1371/journal.pone.0277146)

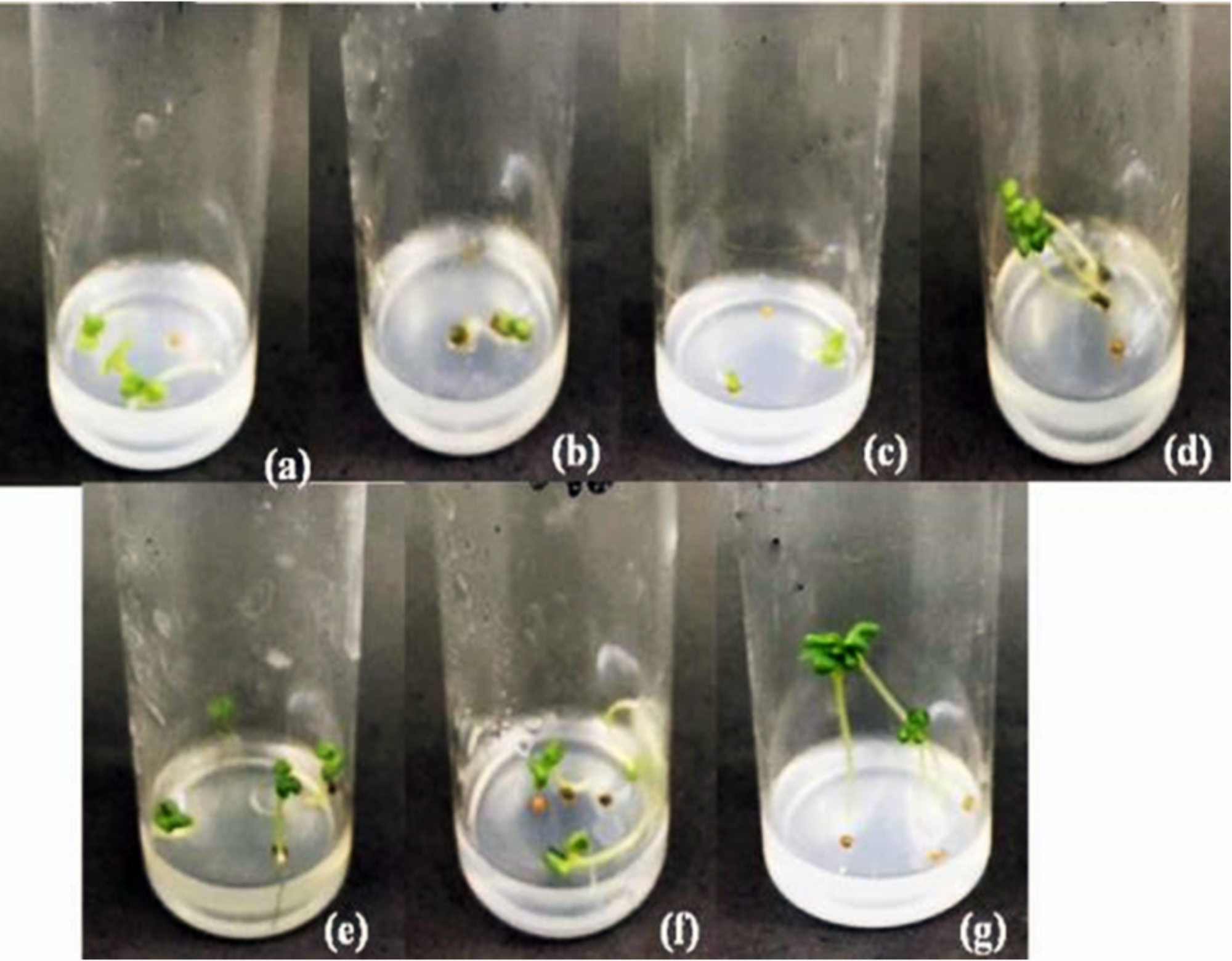

Supplement: S1 Fig — (a) 0.6 mM FA; (b) 0.8 mM FA; (c) 1 mM FA; (d) 0.6 mM GA; (e) 0.8 mM GA; (f) 1 mM GA and (g) Aqueous control. Seed germination was significantly inhibited with 0.8 and 1 mM FA and GA. (TIF) [file pone.0277146.s001.tif]

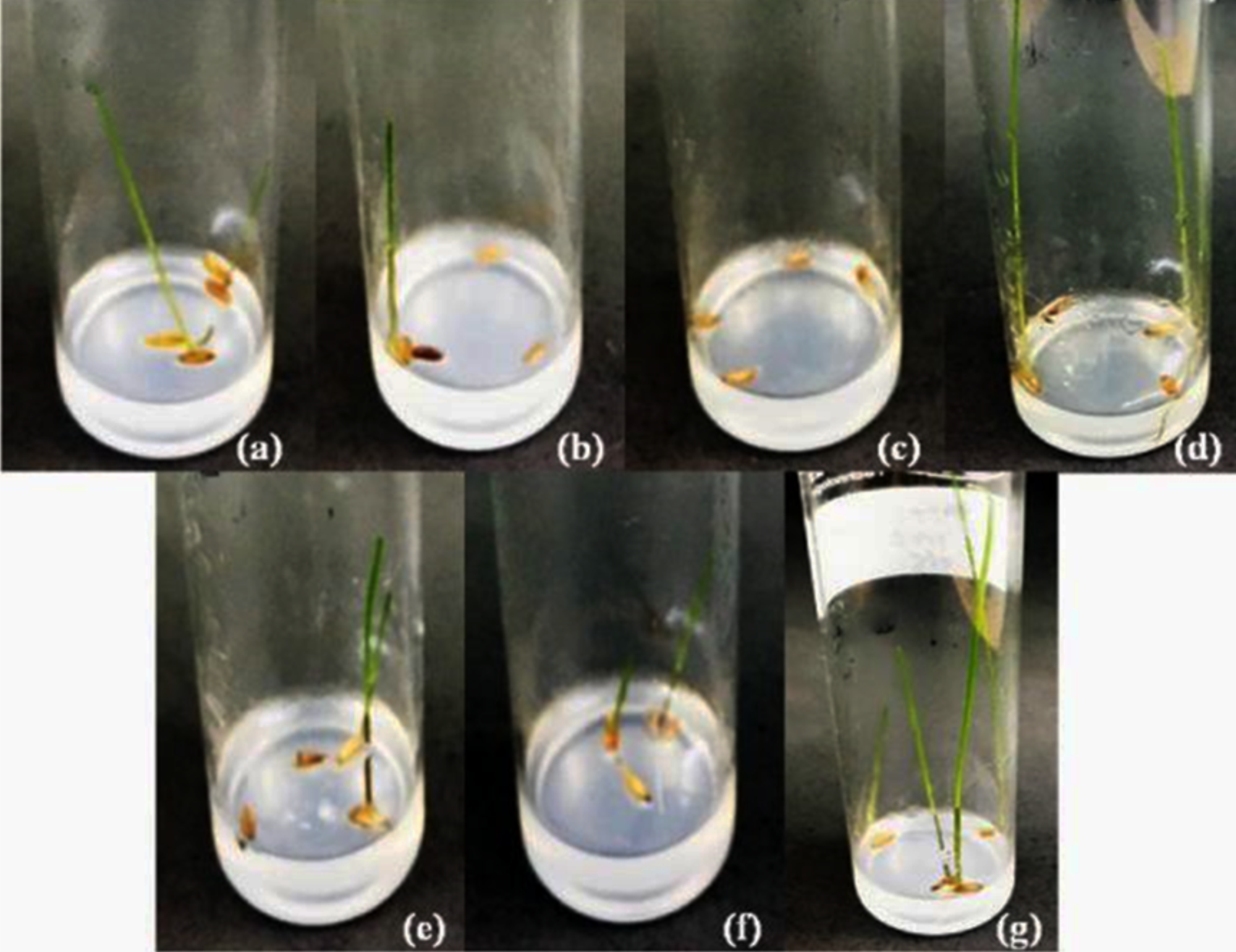

Supplement: S2 Fig — (a) 0.6 mM FA; (b) 0.8 mM FA; (c) 1 mM FA; (d) 0.6 mM GA; (e) 0.8 mM GA; (f) 1 mM GA and (g) Aqueous control. Seed germination was significantly inhibited with 0.8 and 1 mM FA and GA. (TIF) [file pone.0277146.s002.tif]

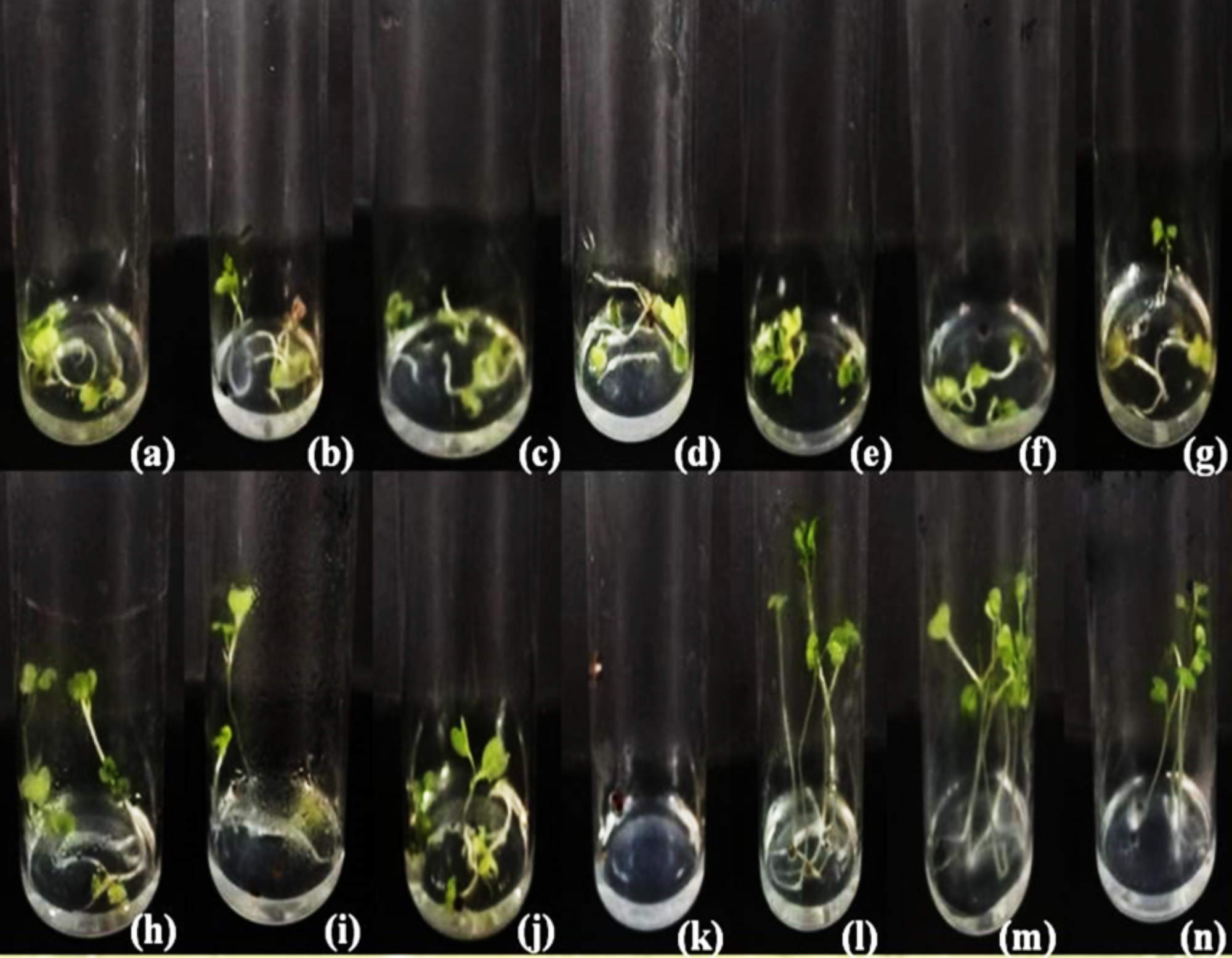

Supplement: S3 Fig — (a) to (l) represent analog 1 to 12 (0.25 mM); (m) 0.25 mM DMSO; (n) aqueous control. Analog 1, 6, 7 and 11 reduced seed germination and wilted seedling, if grown. (TIF) [file pone.0277146.s003.tif]

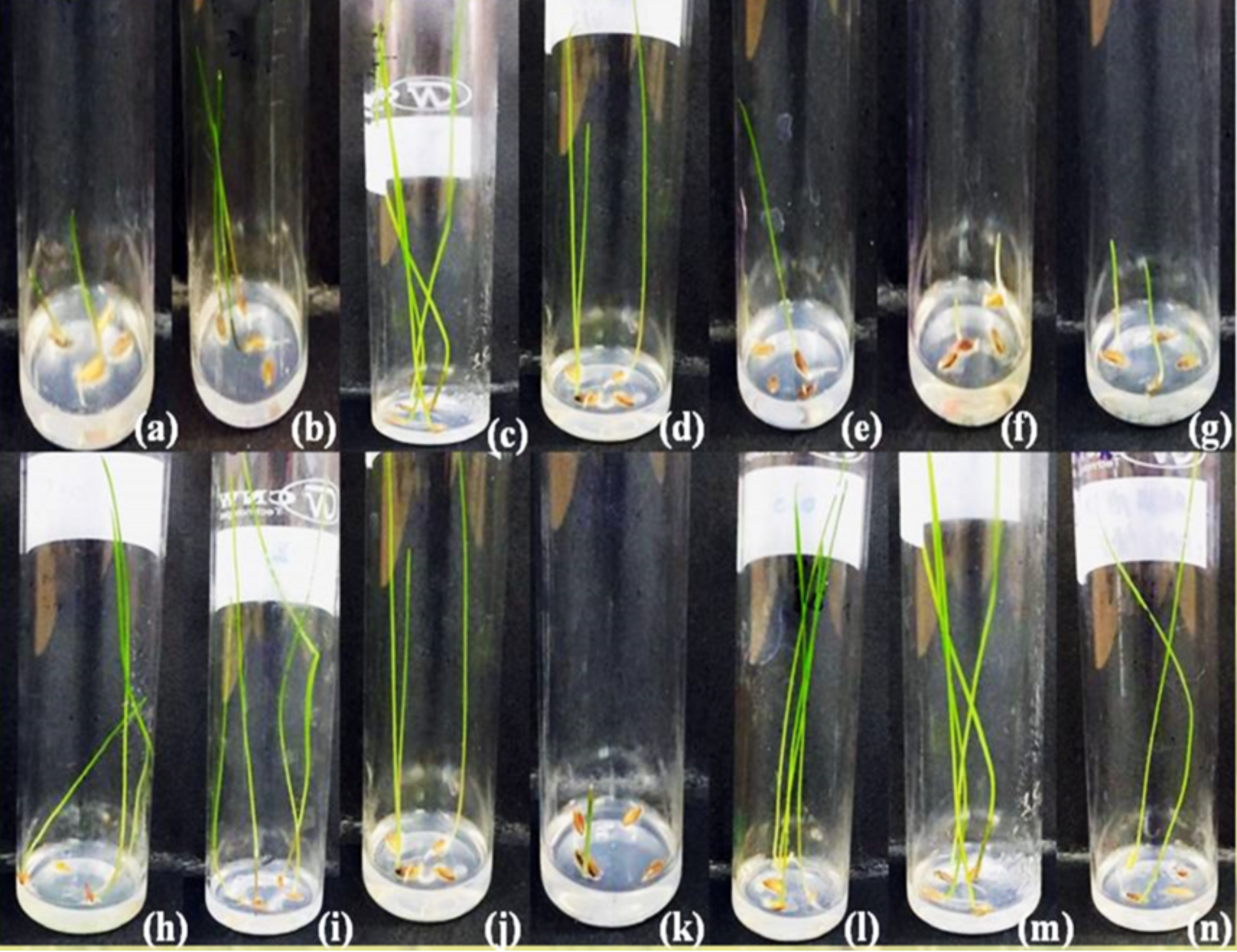

Supplement: S4 Fig — (a) to (l) represent analog 1 to 12 (0.25 mM); (m) 0.25 mM DMSO; (n) aqueous control. Analog 1, 6, 7 and 11 reduced seed germination and wilted seedling, if grown. (TIF) [file pone.0277146.s004.tif]

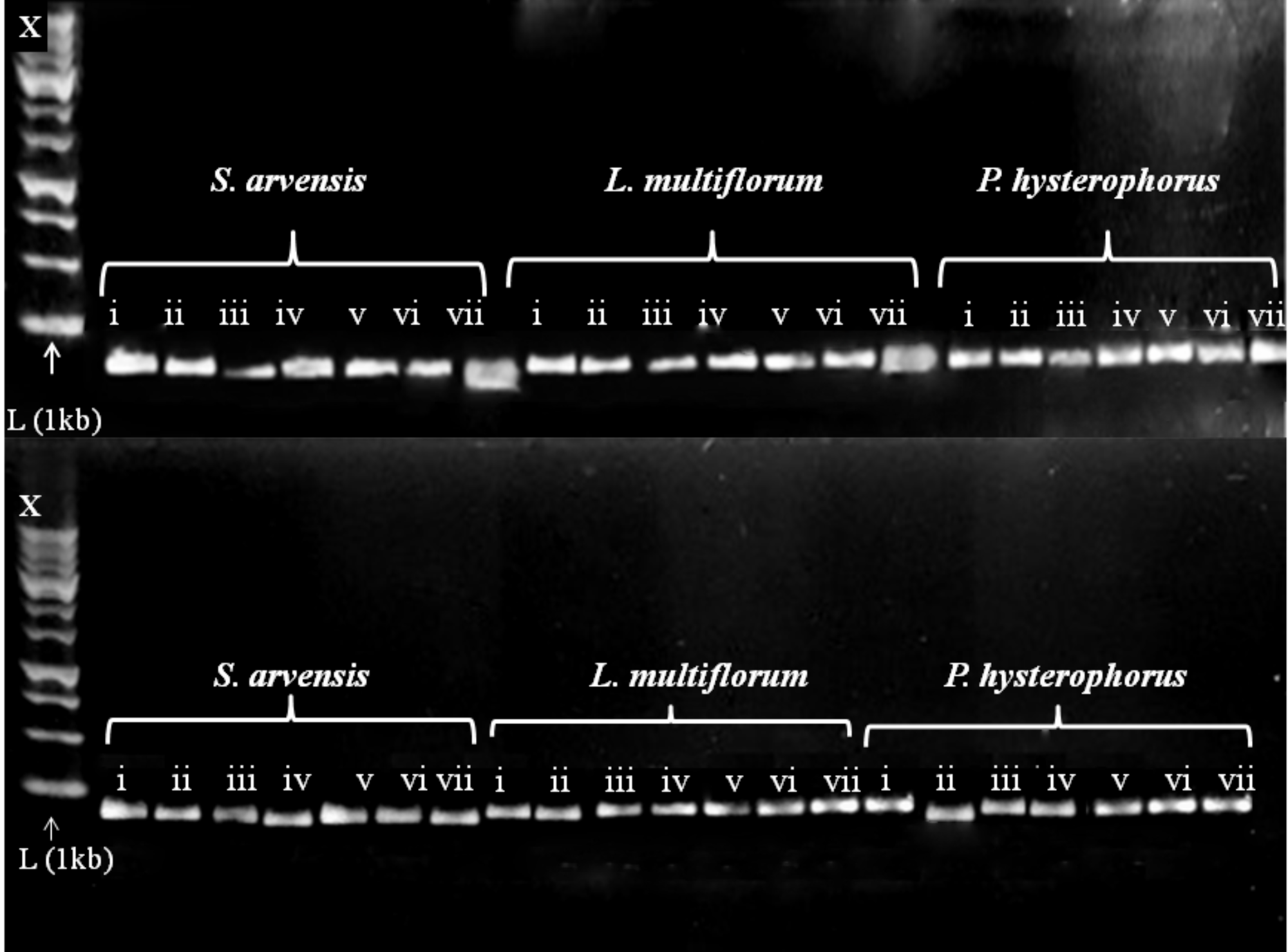

Supplement: S1 Raw image — For each weed; (i) 1mM FA; (ii) 2mM FA; (iii) 3mM FA; (iv) 1mM GA; (v) 2mM GA; (vi) 3mM GA; (vii) aqueous control. (L = 1 kb DNA ladder; X = lane not shown in original manuscript). Gels were visualized and photographed for analysis in gel documentation system (Genosens 1560). (TIF) [file pone.0277146.s005.tif]
