# Supplementary material for: Biochemical analysis, photosynthetic gene (psbA) down–regulation, and in silico receptor prediction in weeds in response to exogenous application of phenolic acids and their analogs
Source: PLoS One. 2023 Mar 23;18(3):e0277146. doi: 10.1371/journal.pone.0277146 (PMC10035924; doi:10.1371/journal.pone.0277146)
Supplement: S1 Table — (DOCX) [file pone.0277146.s006.docx]

| Sample | area | mean | min | max | indent | rawindent |
| --- | --- | --- | --- | --- | --- | --- |
| 1 | 1128 | 67.672 | 0 | 245 | 63890 | 63890 |
| 2 | 1126 | 64.386 | 0 | 246 | 66402 | 66402 |
| 3 | 1108 | 60.602 | 0 | 241 | 57168 | 57168 |
| 4 | 1148 | 61.764 | 0 | 241 | 37736 | 37736 |
| 5 | 1136 | 77.391 | 0 | 255 | 92559 | 92559 |
| 6 | 1104 | 64.975 | 0 | 245 | 71732 | 71732 |
| 7 | 1056 | 56.341 | 0 | 250 | 59496 | 59496 |
| 8 | 1056 | 61.743 | 0 | 248 | 65201 | 65201 |
| 9 | 1160 | 68.873 | 0 | 246 | 66118 | 66118 |
| 10 | 1160 | 62.384 | 0 | 253 | 59889 | 59889 |
| 11 | 1064 | 69.286 | 0 | 255 | 59863 | 59863 |
| 12 | 1160 | 61.365 | 0 | 255 | 58910 | 58910 |
| 13 | 1152 | 65.111 | 0 | 255 | 75008 | 75008 |
| 14 | 1056 | 62.723 | 0 | 255 | 87355 | 87355 |
| 15 | 1152 | 77.984 | 0 | 255 | 89837 | 89837 |
| 16 | 1080 | 61.264 | 0 | 245 | 68572 | 68572 |
| 17 | 1145 | 62.893 | 0 | 248 | 68542 | 68542 |
| 18 | 1155 | 60.987 | 0 | 248 | 87452 | 87452 |
| 19 | 1091 | 63.547 | 0 | 255 | 72485 | 72485 |
| 20 | 1138 | 63.438 | 0 | 245 | 94357 | 94357 |
| 21 | 1161 | 62.349 | 0 | 255 | 78572 | 78572 |

**S1 Table. Quantification data of reference gene (tubulin) determined by Image J software.**
