# Supplementary material for: Biochemical analysis, photosynthetic gene (psbA) down–regulation, and in silico receptor prediction in weeds in response to exogenous application of phenolic acids and their analogs
Source: PLoS One. 2023 Mar 23;18(3):e0277146. doi: 10.1371/journal.pone.0277146 (PMC10035924; doi:10.1371/journal.pone.0277146)
Supplement: Attachment — Submitted filename: Response to Reviewers (1).docx [file pone.0277146.s007.docx]

Response to Reviewers

On behalf of all authors, I am highly thankful to the reviewers for their valuable time they have given to improve this manuscript. We have thoroughly reviewed the comments and improved the manuscript accordingly. Below are the reviewer’s comments (black marked) and responses to the comments (red marked)

Journal requirements:

When submitting your revision, we need you to address these additional requirements.

1.  Please ensure that your manuscript meets PLOS ONE's style requirements, including those for file naming.

Response 1. Manuscript has been checked thoroughly and we followed the journal format requirements.

2. Thank you for stating the following financial disclosure:

“YES- Pakistan Science Foundation provided the research funding through grant no. PSF-NSLP 663.”

Please state what role the funders took in the study.  If the funders had no role, please state: "The funders had no role in study design, data collection and analysis, decision to publish, or preparation of the manuscript."

If this statement is not correct you must amend it as needed.

Please respond by return e-mail so that we can amend your financial disclosure and competing interests on your behalf.

Response 2. The mentioned statement about financial disclosure is correct and is also added in the manuscript. Please see page 24, line 513 – 516.

3. Thank you for stating the following in your Competing Interests section:

“No authors have competing interests.”

Please complete your Competing Interests on the online submission form to state any Competing Interests. If you have no competing interests, please state "The authors have declared that no competing interests exist.", as detailed online in our guide for authors at http://journals.plos.org/plosone/s/submit-now

 This information should be included in your cover letter; we will change the online submission form on your behalf.

Response 3. "The authors have declared that no competing interests exist." also added in the main manuscript. Please see page 24, line 511.

4. PLOS ONE now requires that authors provide the original uncropped and unadjusted images underlying all blot or gel results reported in a submission’s figures or Supporting Information files. This policy and the journal’s other requirements for blot/gel reporting and figure preparation are described in detail at https://journals.plos.org/plosone/s/figures#loc-blot-and-gel-reporting-requirements and https://journals.plos.org/plosone/s/figures#loc-preparing-figures-from-image-files. When you submit your revised manuscript, please ensure that your figures adhere fully to these guidelines and provide the original underlying images for all blot or gel data reported in your submission. See the following link for instructions on providing the original image data: https://journals.plos.org/plosone/s/figures#loc-original-images-for-blots-and-gels.

In your cover letter, please note whether your blot/gel image data are in Supporting Information or posted at a public data repository, provide the repository URL if relevant, and provide specific details as to which raw blot/gel images, if any, are not available. Email us at plosone@plos.org if you have any questions.

Response 4. Raw files of gel images and data are uploaded as supporting information. Captions for supporting information are added at the end of manuscript. Please see page 35, line 778 – 800.

5. Please upload a copy of Figures S1 and S2, to which you refer in your text on page 13. If the figure is no longer to be included as part of the submission please remove all reference to it within the text.

Response 5. Fig S1 – S4 are uploaded individually as supporting information. Captions for all supplementary figures are added. Please see page 35, line 778 – 800.

6. Please review your reference list to ensure that it is complete and correct. If you have cited papers that have been retracted, please include the rationale for doing so in the manuscript text, or remove these references and replace them with relevant current references. Any changes to the reference list should be mentioned in the rebuttal letter that accompanies your revised manuscript. If you need to cite a retracted article, indicate the article’s retracted status in the References list and also include a citation and full reference for the retraction notice.

Response 6. Needful done. Reference list has been reviewed thoroughly and it is according to the Journal guidelines. There are no retracted articles cited in the manuscript.

**Reviewer #1:**	The authors studied the efficacy of the ferulic acid and gallic acid to three prevalent herbicide-resistant weed species. In silico analysis found new receptors of ferulic acid and gallic acid in psbA protein. They also found four analogs for developing plant based formulation to suppress weed growth. However, there several items should be addressed.

1. I cannot read the supporting information.

Response 1. Needful done. Supporting information including Fig S1 – S4 have been upload individually. Captions for all the supporting information are added at the end of the revised manuscript. Please see page 35, line 778 – 800.

2. Figure 9 B, it is strange to me that the effect of analog 1 is a, it seems similar with analog 6,7 and 11.

Response 2. The valuable comment is highly acknowledged. The figure has been rechecked and corrected. Please see updated Fig 9.

3. The effect of ferulic acid, gallic acid and the analogs to the germination of P. hysterophorus is missing.

Response 3. *In vitro* phytotoxicity of compounds was investigated against *S. arvensis* and *L. multiflorum* as representatives of dicot and monocot species. These two species grow easily and rapidly under similar *in vitro* conditions. [Please see:

- Javaid, M. M., Mahmood, A., Alshaya, D. S., AlKahtani, M. D., Waheed, H., Wasaya, A., ... & Fiaz, S. (2022). Influence of environmental factors on seed germination and seedling characteristics of perennial ryegrass (Lolium perenne L.). *Scientific Reports*, *12*(1), 1-11.
- Singh, A., Mahajan, G., & Chauhan, B. S. (2022). Germination ecology of wild mustard (Sinapis arvensis) and its implications for weed management. *Weed Science*, *70*(1), 103-111.]

In our earlier research it was observed that germination of *S. arvensis* and *L. multiflorum* took up to 3 and 5 days respectively while *P. hysterophorus* germinated between 10 to 15 days.

[Please see: Anwar, S., Naseem, S., Karimi, S., Asi, M. R., Akrem, A., & Ali, Z. (2021). Bioherbicidal Activity and Metabolic Profiling of Potent Allelopathic Plant Fractions Against Major Weeds of Wheat—Way Forward to Lower the Risk of Synthetic Herbicides. *Frontiers in plant science*, 333.]

Thus preliminary phytotoxicity and dose optimization of phenolic acids and analogs was performed against one dicot and monocot species each to determine their earlier responses for the purpose of standardization of experiments.

4. Figure 8, There is no effect of analog 1 to the germination of weed species. So, not four but three analogs have been identified.

Response 4. In principle we agree with this statement. Although analog 1 did not show significant inhibitory effect on the germination percentage but it was observed to have negative impact on seedling growth, since the seeds germinated, but could not grow well and wilted within the time of data collection. Statements have been amended as per suggestion by the worthy reviewer in the manuscript. Please see page 18, 19; line 378, 391 – 393. Further, biochemical analysis of investigated analogs against weeds also depicted negative results, including analog 1. Please see line 395 – 398.

**Reviewer #2:**	In general, the manuscript is sound and the data are well presented! Based on data presented the conclusions are well sustained! Hence, I recommend publication of the manuscript after a minor revision!

The choice of a semiquantitative PCR technique for determination of psbA transcripts is not ideal! A Real-time RT-PCR approach would have been more appropriate and is recommended. However if this is not possible please include in manuscript or supporting materials the quantitation of the reference gene. It is really important that the expression of this gene should not be influenced by your treatments.

The manuscript should also be carefully read for minor language and syntax errors!

We appreciate the comment. Unfortunately, real-time PCR could not be performed, but the semi-quantitative PCR was performed with ultimate care and replicated to obtain valid results. Quantification of reference gene (tubulin) was done. Its expression was found to be uninfluenced by application of treatments, which can be been seen by the banding patterns and their quantification. The table for tubulin quantification along with the analysis of psbA gene is added as supporting information. Please see S1 Table. The manuscript has been carefully proofread for minor errors.
